# Efficacy of acupuncture and laser acupuncture in temporomandibular disorders: a systematic review and meta-analysis of randomized controlled trials

**DOI:** 10.1186/s12903-023-03806-1

**Published:** 2024-02-03

**Authors:** Fabrizio Di Francesco, Giuseppe Minervini, Yuliia Siurkel, Marco Cicciù, Alessandro Lanza

**Affiliations:** 1 Multidisciplinary Department of Medical, Surgical and Dental Sciences, Campania University Luigi Vanvitelli, Naples, Italy; 2 Saveetha Dental College and Hospitals, Saveetha Institute of Medical and Technical Sciences (SIMATS), Saveetha University, Chennai, Tamil Nadu, India; 3 International European University School of Medicine, Akademika Hlushkova Ave, 42В, Kyiv, 03187 Ukraine; 4 Department of General Surgery and Medical-Surgical Specialties, School of Dentistry, University of Catania, Catania, 95124 Italy

**Keywords:** Acupuncture, Temporomandibular disorders, TMD, Laser

## Abstract

**Objective:**

The aim of this study is to perform a qualitative and quantitative analysis of the scientific literature regarding the use of acupuncture and laser acupuncture in the treatment of pain associated with temporomandibular disorders (TMDs). The aim of this article was to assess the clinical evidence for acupuncture and laser acupuncture therapies as treatment for temporomandibular joint disorder (TMD).

**Materials and methods:**

This systematic review includes randomized clinical trials (RCTs) of acupuncture and laser acupuncture as a treatment for TMD compared to other treatments. Systematic searches were conducted in 3 electronic databases up to July 2023; PubMed, EMBASE, and SCOPUS databases. All RCTs of acupuncture for TMD were searched without language restrictions. Studies in which no clinical data and complex interventions were excluded. The Cochrane risk of bias tool (RoB 2) tool was employed to analyze randomized controlled trials. A Meta-analysis was performed in order to investigate a quantitative analysis comparing acupuncture and laser acupuncture to placebo.

**Results:**

A total of 11 RCTs met our inclusion criteria. The findings show that acupuncture is short-term helpful for reducing the severity of TMD pain with muscle origin. Meta-analysis revealed that the Acupuncture group and Laser Acupuncture group had a higher efficacy rate than the Placebo control group, showing a high efficacy of Acupuncture and Laser Acupuncture group in the treatment of temporomandibular.

**Conclusions:**

In conclusion, our systematic review demonstrate that the evidence for acupuncture as a symptomatic treatment of TMD is limited. Further rigorous studies are, however, required to establish beyond doubt whether acupuncture has therapeutic value for this indication. However high efficacy of Laser Acupuncture in the treatment of temporomandibular disorders was reported.

## Introduction

Temporomandibular disorders (TMDs) refer to various conditions affecting the temporomandibular joint, masticatory muscles, and contiguous tissues components [1]. Different types of painful TMDs are encountered: myogenous or muscle-generated pain; arthrogenous or joint generated pain; or both [2, 3]. According to De Rossi et al., [2] between 90% and 95% of TMD patients have facial pain of muscular origin without identifiable structural causes. Among the painful TMD of muscular origin, the most frequent is myofascial pain (MP). At present, the therapeutic management of TMD is approached using a medical multidisciplinary model, and the treatment options range from conservative, noninvasive therapeutic measures to more aggressive treatment interventions [4]. However, in most of the mild and moderate cases of TMD, a significant clinical improvement can be obtained with conservative therapeutic modalities [5–14]. Among the nonsurgical treatment procedures for TMD are self-management measures (home care), occlusal splints, medication, cognitive-behavioral techniques, and various forms of physiotherapeutic treatment [15].

Acupuncture is an increasingly used treatment modality for the therapeutic management of pain symptoms [16]. A systematic review performed by Wen et al. showed that the majority of the studies documented positive results [17–24]. However, the main conclusion obtained from that meta-analysis was that the methodologic quality of the studies analyzed required more evidence. For this reason, the investigators concluded that the effects of acupuncture for chronic pain are doubtful.

Recently, laser acupuncture therapy (LAT) has been proposed as an alternative to conventional acupuncture therapy to eliminate the need for needle insertion. In this way, low-intensity laser light is employed for stimulating the traditional acupuncture points, and so the procedure is simple, non-aggressive, painless, and inherently safer than needle acupuncture therapy [25–42]. However, studies on laser acupuncture regarding this specific theme are scarce, in spite of the reported advantages of this technique over the use of needles.

The aim of this study is to perform an analysis that evaluates the quality of the studies and the effectiveness of acupuncture and laser acupuncture treatments in relieving painful TMD symptomatology.

## Materials and methods

### Eligibility criteria

All documents based were selected on the following Population, Intervention, Comparator and Outcomes (PICO) model:

- (P) Participants consisted of human subjects with Temporomandibular disorders.
- (I) The Intervention consisted of acupuncture and laser acupuncture treatments.
- (C) The Comparison consisted of acupuncture and laser acupuncture treatments compared to Placebo treatment.
- (O) Outcome: To assess the efficacy of treatments in terms of post-operative clinical parameters such as pain intensity or pain relief measured by visual analogue scale (VAS), verbal scale, numerical scale (0–10 point) or pressure algometer, and maximum inter-incisal mouth opening (MO) and muscle tenderness.

**Fig. 1:**
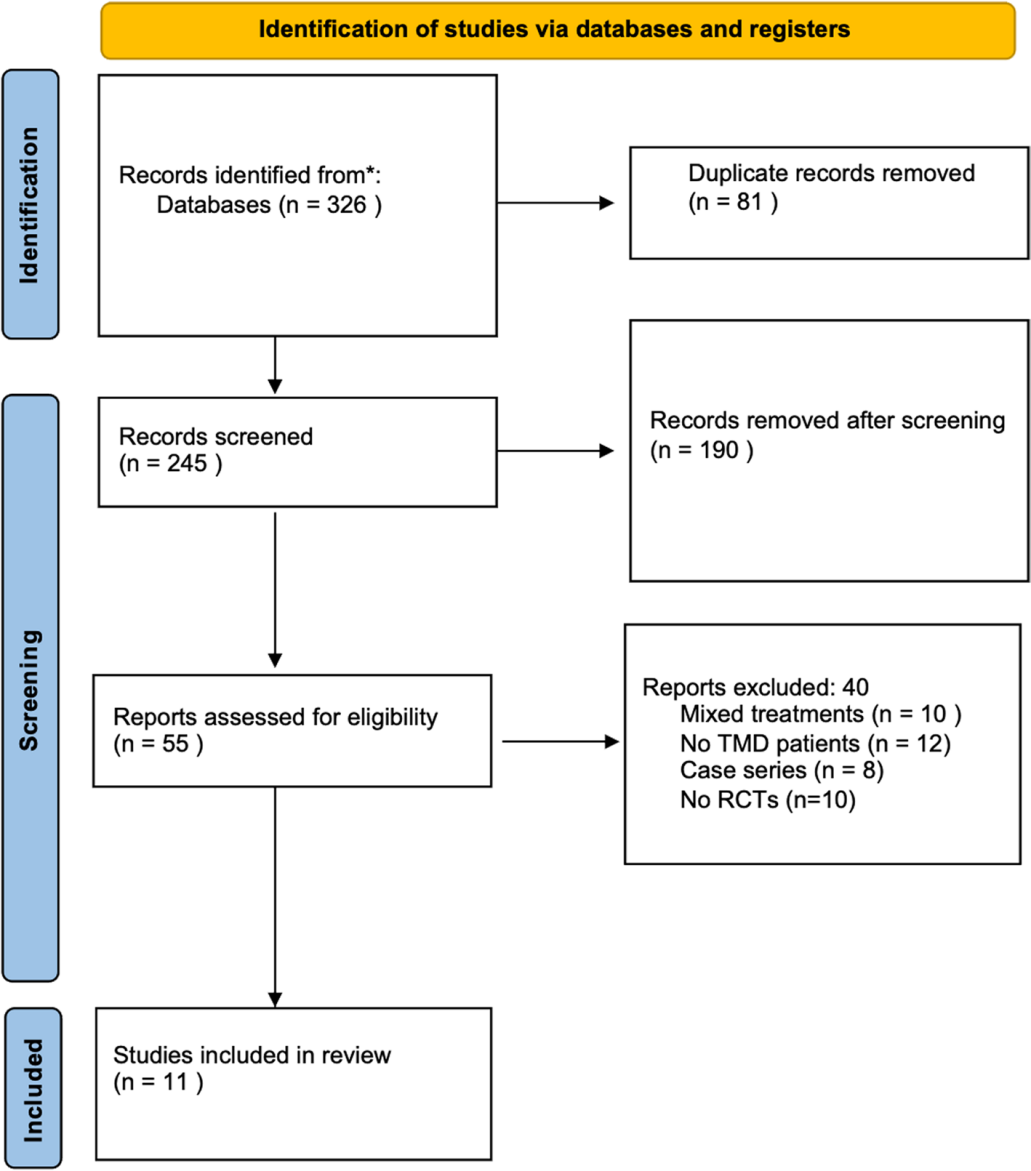
The flowchart of data selection

When considering the studies to include in this analysis, 2 groups were identified and selected based on the criteria required for the systematic review. The inclusion criteria for the systematic review were the following: (1) only randomized controlled trials (RCTs); (2) studies performed on patients diagnosed with TMD who suffered painful symptoms; (3) studies in which manual acupuncture and/or laser acupuncture were used; (4) studies published in scientific journals between 2000 and 2023; (5) studies performed on adult patients (more than 18 y old); (6) studies in which the technical aspects of the acupuncture and/or laser acupuncture treatments were described, for example, location of the acupuncture points and duration of treatment; (7) studies in which the control group (CG) received other treatment, i.e. a placebo treatment, a drug therapy, laser regular TMD treatment, or decompression splint; (8) English language publications. All studies that had multiple therapeutic interventions applied in the study groups or those that did not fulfill the criteria of a RCT were excluded. The Fig. 1 shows the methodologic development of the review and the studies excluded. One of the following outcome measures was required for inclusion: pain intensity or pain relief in TMJ measured by visual analogue scale (VAS), verbal scale, numerical scale (0–10 point) or pressure algometer. Other clinically important outcomes included measured maximum inter-incisal mouth opening (MO) and muscle tenderness.

### Search strategy

Articles were researched from 2000 using the following databases: MEDLINE, EMBASE and SCOPUS. The terms used for the search were derived from a combination of the following words: ‘‘acupuncture,’’ ‘‘laser acupuncture,’’ ‘‘temporomandibular joint dysfunction,’’ ‘‘temporomandibular disorders,’’ ‘‘orofacial pain,’’ ‘‘myofascial pain,’’ ‘‘facial pain,’’ ‘‘pain’’, and ‘‘randomized controlled trial.’’ The first analysis of the studies was based on the information provided by the summary, the title, and the key words. This process was performed by 2 independent reviewers. The articles selected for this analysis were evaluated in detail using the complete text in the analytic phase. This systematic review followed the Preferred Reporting Items for Systematic Reviews (PRISMA) guidelines and the Cochrane Handbook for Systematic Reviews of Interventions. PROSPERO’s International Prospective Register of Systematic Reviews lists the systematic review protocol under the provisional accession number 45646.

### Data extraction

Two reviewers (F.D. and G.M.) independently extracted the data from the included studies using a tailored data extraction on an Excel sheet. A third reviewer reached a consensus in case of disagreement (A.L.).

The following data were extracted: First author, year of publication, design/ types of TMD, intervention and control groups, main outcomes and intergroup differences. The following data were extracted and included in Table 1. Two authors read all articles independently, and the data were compared and contextualized.

**Table 1:** Features of included studies. AT: acupuncture; MO: mouth opening; TMD: temporomandibular joint disorder; VAS: visual analogue scale (100 mm scale); NRS: numeric rating scale (0–10 point, 11point scale); RCT: randomized clinical trial; n.r.: not report;

First author (year)	Design/ Types of TMD	Intervention group	Control group	Main outcomes	Intergroup differences
Madani (2020) [43]	RCT/Muscular	Laser AT (30 min,once,*n* = 15)	Low-level laser therapy, *n* = 15 and placebo, *n* = 15	VAS MO	*P* < 0.05 *P* < 0.05
Gonzales-Perez (2015) [44]	RCT/Muscular	AT (30 min, once/ week for 3 weeks ,*n* = 24)	Combination drug therapy (methocarbamol and paracetamol, *n* = 24)	VAS MO	*p* = 0.016 *p* = 0.011
Ferreira (2013) [45]	RCT/Combined	Laser AT (n.r,once/week 12 sessions, *n* = 27)	Sham laser associated with occlusal splint therapy, *n* = 26	VAS	*P* = 0.001
Vicente-Barrero (2012) [46]	RCT/Combined	AT (30 min,15 session for 5 weeks ,*n* = 10)	Decompression splint, *n* = 10	VAS pressure algometer	*P* = 0.06 *P* = 0.06
Katsoulis (2010) [47]	RCT/Muscular	Laser AT (15 min, twice/week, 6 sessions for 3 weeks,*n* = 4)	Sham laser (acupoint, non-activated sham laser, 1session = 15 min each, 2 times/week, 6 sessions, *n* = 3	VAS Verbal scale (pain free %)	*P* < 0.05 *P* < 0.05
Simma (2009) [48]	RCT/Muscular	AT (n.r, once, *n* = 11)	Sham laser (acupoint, non-activated sham laser, n.r., once, *n* = 12)	VAS MUSCLE TENDERNESS	*P* = 0.40 *P* = 0.10
Shen (2009) [49]	RCT/Muscular	AT (15 min,once, *n* = 16)	Sham AT (1 cm distal to LI4, non-penetrating, 15 min once, *n* = 12)	VAS NSR	*P* = 0.24 *P* = 0.84
Smith (2007) [50]	RCT/Combined	AT (20 min,6 times for 3 weeks, *n* = 15)	Sham AT (Park sham needle, acupoint, non- penetrating skin, 20 min once, 6 times for 3 weeks, *n* = 12)	VAS MO MUSCLE TENDERNESS	*P* = 0.10 *P* = 0.10 *P* = 0.12
Shen (2007) [51]	RCT/Muscular	AT (15 min,once, *n* = 9)	Sham AT (1 cm distal to LI4, non-penetrating, 15 min once,*n* = 6	VAS NSR	*P* = 0.06 *P* = 0.22
Schmid-Schwap (2006) [52]	RCT/Combined	AT (20 min,6 times for 3 weeks, *n* = 11)	Sham laser (acupoint, non-activated sham laser, 20 min once, *n* = 12)	VAS MO MUSCLE TENDERNESS	*P* = 0.04 *P* = 0.10 *P* = 0.002
Goddard (2002) [53]	RCT/Muscular	AT (30 min,once,*n* = 10)	Sham AT (30 min once, nonacupoint, penetrating skin, 2–4 mm depth, *n* = 8)	VAS	*P* = 0.27,

### Statistical analysis

The pooled analyses were performed using the soft- ware Review Manager version 5.2.8 (Cochrane Collaboration, Copenhagen, Denmark; 2014). Only studies reporting data referring to the efficacy of Acupuncture and Laser Acupuncture group in temporomandibular disorders compared to a Placebo control group were included in the quantitative analysis.

The Odds Ratio between the two groups was measured. Heterogeneity among studies was evaluated by means of the Higgins Index (*I2*) and the chi- square test and classified as follows: low heterogeneity (< 30%), medium heterogeneity (30–60%), and high heterogeneity (> 60%).

## Results

### Study characteristics

The flowchart of data selection is shown in Fig. 1. A total of 326 articles were found in the electronic search. After all titles were checked, 81 duplicates were removed, and 245 articles were selected for abstract reading. Then, the analysis of the abstracts excluded articles that clearly did not satisfy the eligibility criteria. Therefore, 55 full-text articles were identified. Finally, 11 full-text articles satisfied the inclusion criteria. Features of the included studies are reported in Table 1.

The diagnostic methods of TMD used were five trials [51–55] diagnosed the subjects by Research diagnosed criteria (RDC/ TMD) [50], the others did not report which diagnostic systems were used [43–47, 49]. In all studies, for the types of TMD, seven studies were muscular type [56–62], none was articular type, and four were combined type of both [63–66]. Of the 11 total studies, six studies comparatively tested needle acupuncture against penetrating sham acupuncture [67], combination drug therapy [44], non- penetrating sham acupuncture [49–51], or sham laser acupuncture [52, 55] ,whilst the remaining study tested laser acupuncture against sham laser acupuncture [43, 45, 47] and low-level laser acupuncture [43]. Five of the studies only evaluated the effects of short-term acupuncture treatment [44, 50, 52, 53, 55].The remaining 2 studies evaluated the short- and long-term effects, as they re-evaluated the patients at 6 months [46, 49] and 1 year [46]after the study was completed. In terms of the technical aspects of the acupuncture treatment used in the RCT, the duration of each session varied between 15^48 and 51^, 20^53,54^ and 30 min. The employed acupuncture points were located in specific anatomic areas of the cranio-cervical-mandibular area (ST 5 [51, 55], ST 8 [50, 52, 53], ST1 [44–46, 49], ST7 [43], ST29 [47]. Only six [43–46, 49, 50] of the studies described how deep the needles were inserted. This insertion depth varied from to 30 mm.

All eleven RCTs reported the results in terms of pain intensity by VAS. VAS scale on subjective pain in patients was evaluated in same region in all studies, where were the main area surrounding the TMJ, generally face, jaw and masseter muscle. The time for evaluating VAS scale was immediately after treatment in 6 studies [43, 46, 49–53, 55], the other ones evaluated it after 3 months [44, 45] 16 weeks [47].

Furthermore, four and three RCTs reported the outcomes in terms of mouth opening [43, 44, 50, 52] and muscle tenderness [50, 52, 55] respectively.

### Main findings

Six and three trials showed favorable effects of acupuncture [44, 49, 51–53, 55] or laser acupuncture [43, 45, 47], respectively, whilst the others did not [46, 50].

The findings show that acupuncture is short-term helpful for reducing the severity of TMD pain with muscle origin. It is also important to note that in the study performed by Goddard et et al. [55] ,the control group. However, there was a substantial statistical difference between the pretreatment and posttreatment phases of such an acupuncture treatment. Additionally, some of the RCTs examined demonstrated that individuals with TMDs of muscular origin did have improvements in their interincisal opening and overall masticatory function [44, 50, 51, 55].

Furthermore, the three included RCTs comparing LAT and Placebo [43, 45, 47], showed statistically differences in terms of Vas scale ( *P* < 0.05), highlighting an efficacy of LAT in the treatment of temporomandibular disorders.

Madani et al. [43] showed interesting data comparing both LAT and placebo, and LAT and Low-level laser therapy (LLLT). In comparison to the placebo group, the VAS ratings, the masseter, the temporal tendon, and the insertion of the internal pterygoid muscles, as well as the overall pain intensity, were all considerably reduced in the LLLT and LAT groups. The Authors [43] reported that LAT could be suggested as a suitable alternative to LLLT for patients suffering from TMD, as it provided effective results over a shorter treatment duration.

Furthermore, Ferreira et al. [45]. showed that after three months of treatment, laser acupuncture was effective in getting rid of all temporomandibular and myofascial pain symptoms, and it sped up the healing process compared to a placebo. After three months of treatment, the active therapeutic laser acupuncture is responsible for a higher percentage of patients experiencing symptom remission. The Authors16 concluded that laser acupuncture is a safe, non-invasive, and efficient therapy option for individuals who have undergone conservative treatment since it reduces the chronic pain related to TMD and has no negative side effects.

### Meta-analysis

The meta-analysis was conducted by fixed model effect because of the low heterogeneity (*I*2 = 0%) between the included studies.

**Fig. 2:**
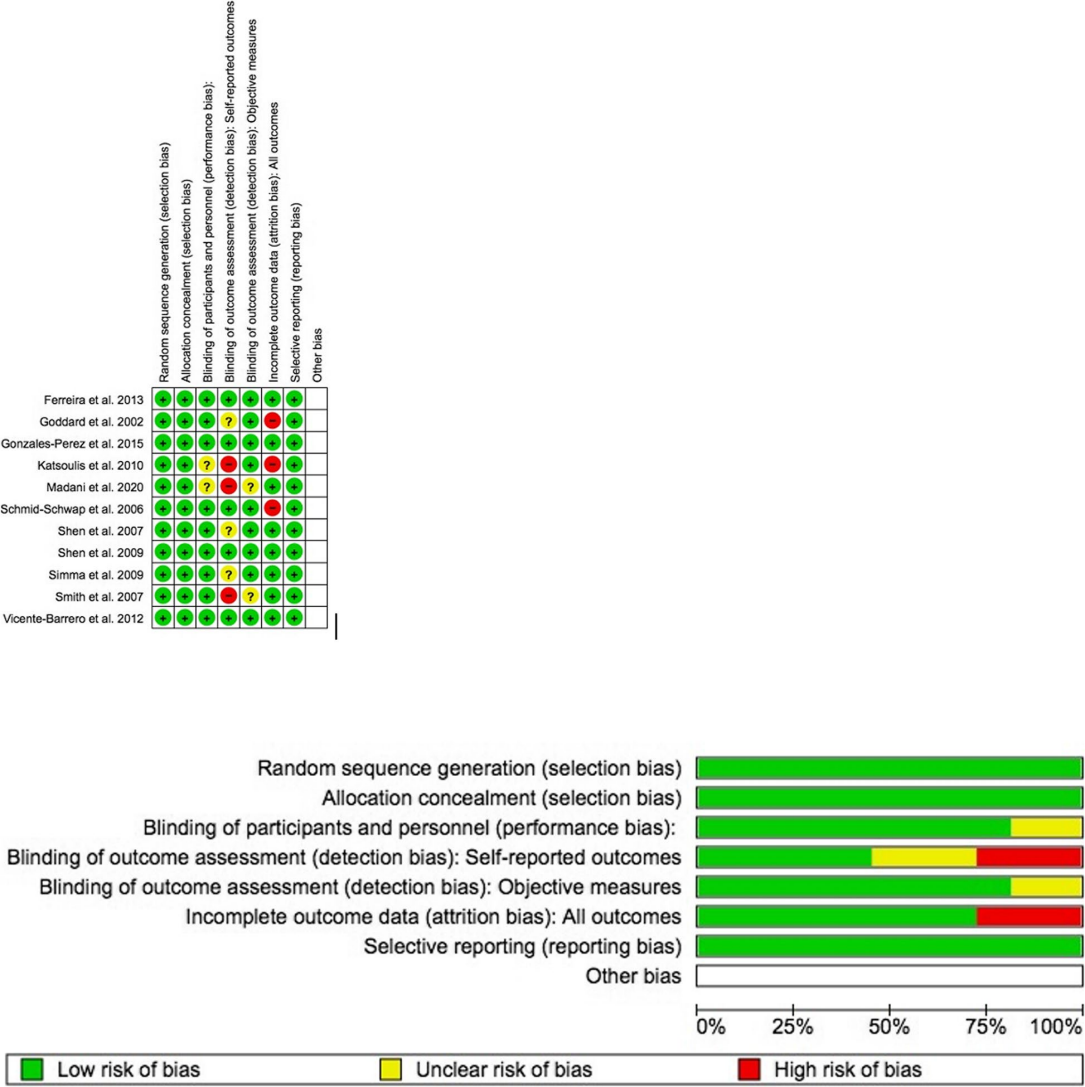
Forest plot of the meta-analysis show the efficacy of Acupuncture Vs Placebo control group

The overall effect, reported in the forest plot (Fig. 2), revealed that the Acupuncture group had a higher efficacy rate than the Placebo control group [6.20; 2.70, 14.24]; showing an efficacy of Acupuncture in the Treatment of temporomandibular disorders.

**Fig. 3:**
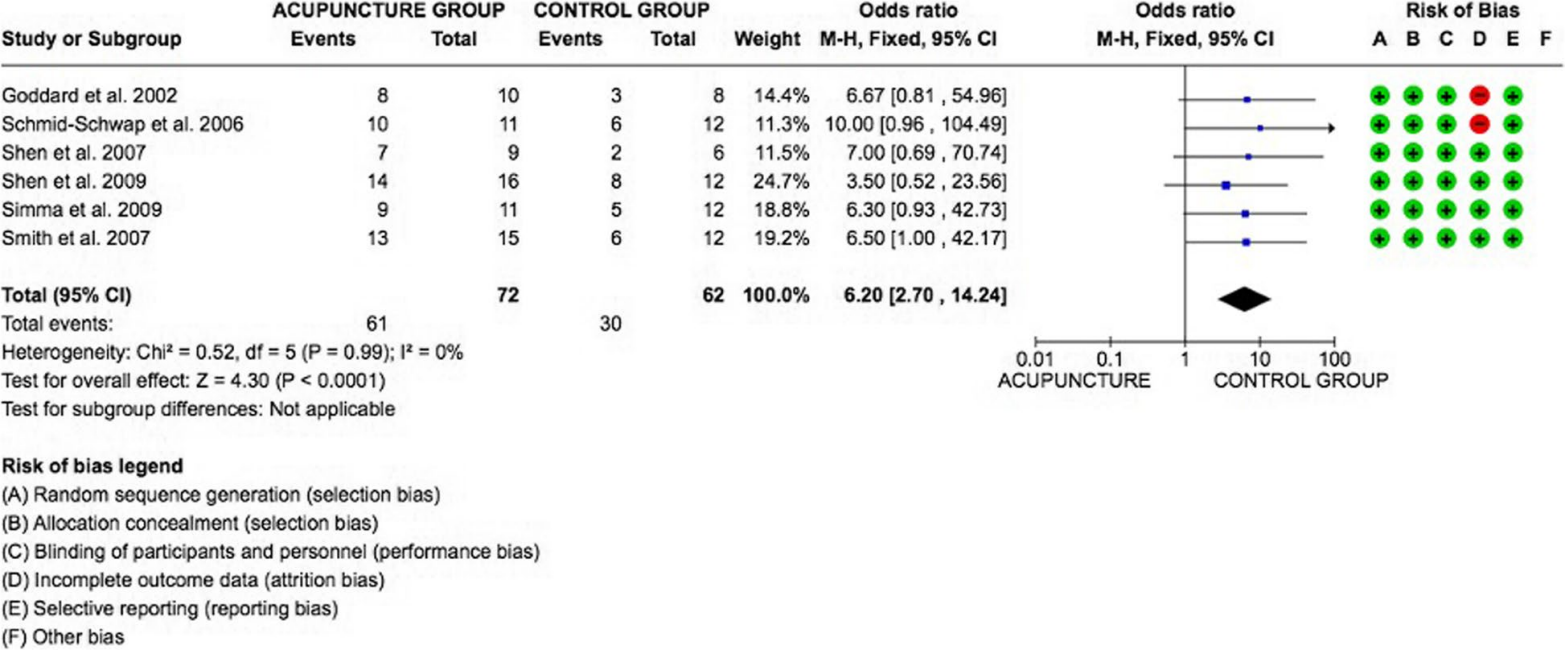
Forest plot of the meta-analysis show the efficacy of Laser Acupuncture Vs Placebo control group

The overall effect, reported in the forest plot (Fig. 3), revealed that the Laser Acupuncture group had a higher efficacy rate than the Placebo control group [14.08; 4.01, 49.47], showing an efficacy of LAT in the treatment of temporomandibular disorders.

### Quality assessment of the studies

**Fig. 4:**
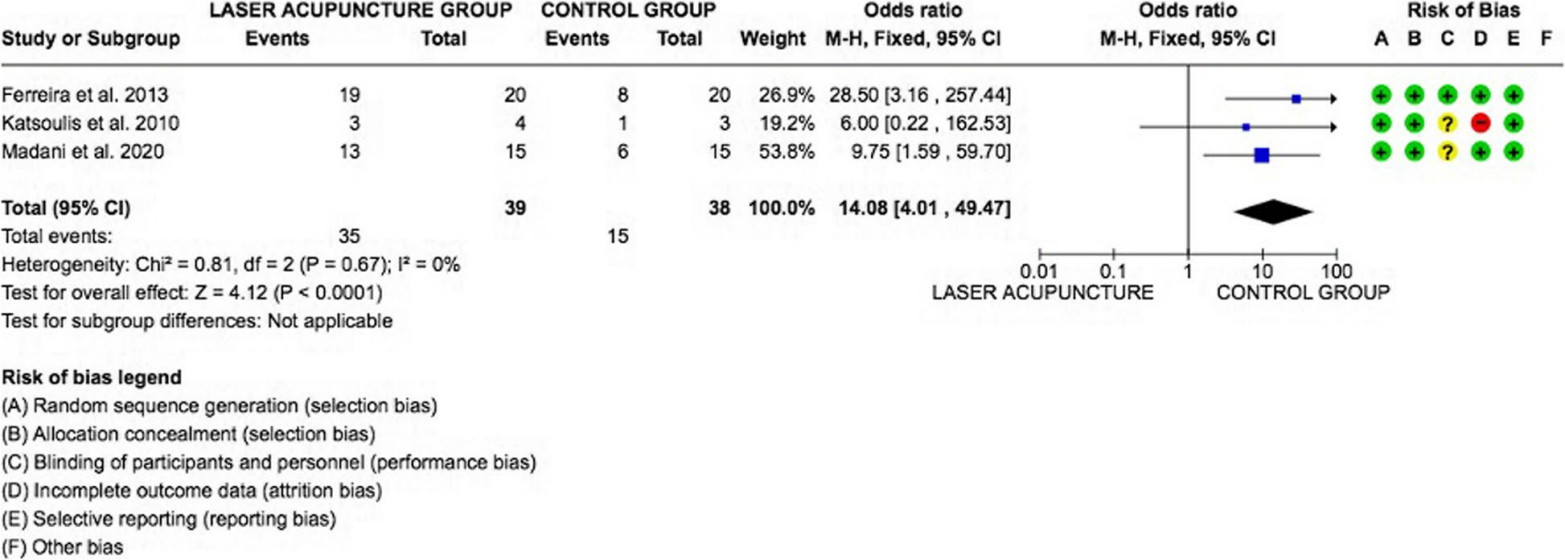
RoB 2 assessment of RCTs. The randomization process reported 100% of the studies with a low risk of bias. Of the studies, 80% excluded a performance bias [44–46, 49–53, 55], but 75% reported all outcome data [43–46, 49–51, 55] and 100% of included trials adequately left out bias in the selection of the reported results [43–47, 49–53, 55]. The overall ranking showed no studies with low risk of bias. Only 4 out of 11 RCT studies were shown to have a low risk of incurring bias [44–46, 51]

Two reviewers individually and independently examined the included studies’ risk of bias (F.D, G.M). In order to analyze randomized controlled trials, the Cochrane risk of bias tool, also known as the RoB 2, was employed [54]. It examines seven bias areas including random sequence generation, allocation concealment, participant and staff blinding, self-reported result assessment blinding, objective measure assessment blinding, incomplete outcome data, selective reporting, and additional biases. Overall, the outcomes of the quality assessment of included RCT studies are reported in Fig. 4. By applying the RoB 2, all the 11 RCTs studies revealed high, low, and unclear risk of bias in some key domains. No studies were shown in the overall rating to have a low risk of bias in accordance with the authors’ definitions [54]. A low risk of bias was identified by the randomization technique for all studies. While 75% of studies disclosed all outcome data, and 100% of the included trials adequately left out bias in the selection of the reported outcomes, only 20% of studies adequately left out performance bias. Only 4 out of 11 RCT trials overall showed a minimal risk of bias.

## Discussion

Only 4 out of 11 RCT trials were proven to have a low risk of bias, necessitating careful interpretation of the study’s findings [44–46, 51]. It is significant to note that the conclusions presented by other researchers in other systematic reviews correspond with the lack of quality seen in other included studies [57, 58, 68]. The remaining 7 papers that were a part of this systematic review are of acceptable quality [45, 46, 49, 51, 53, 55, 69]. The fact that the sample sizes were so small is a problem. It was, however, unquestionably reasonable to include comparisons with a CG employing a placebo when taking into account the beneficial elements of these trials [45, 49, 53, 55, 69]. According to a recent and intriguing study by Goddard et al. [53] ., sham acupuncture can be administered by inserting needles outside of the acupuncture points, and the results did not reveal statistically significant differences between the treatments. This may help to explain why, in addition to the potential placebo effect of acupuncture, there is also a real effect based on the neurophysiology of the needling. In a comparison of the effects of real acupuncture versus sham acupuncture, Goddard et al. [53] reported highly encouraging results on TMJ-related indications and symptoms in the short term. In a recent randomized trial, Shen et al. [51]. showed that genuine acupuncture is more effective than sham acupuncture at treating temporomandibular muscle discomfort even after just one session. Time constraints prevented both a longer term of patient follow-up and the recruitment of a larger sample size. Better and more definitive findings may have been obtained with more subjects and more time [59]. The fact that no study made an attempt to control for the placebo effect was a significant flaw in the way the research for that evaluation was designed. They came to the conclusion that although while every study had suggested that acupuncture may be effective in treating temporomandibular joint dysfunction, there was still a need for additional in-depth research to back up this claim. It is important to emphasize that in the past 10 years, more studies using acupuncture to treat TMD have been published [60–64].The application of AT, LAT, and placebo simultaneously in the management of TMDs is the study’s strength, however the small sample size and brief follow-up time should be viewed as its weaknesses. To further clarify the best course of action for treating patients with TMD, double-blind, randomized controlled trials with a bigger patient sample and lengthy follow-up periods are necessary [65–68]. Future research should examine different laser parameters in LLLT and LAT modalities in order to establish the ideal setting and maximize the physiological benefits of laser therapy for the treatment of TMD, even though Madani et al. [43] found that these treatments were more effective than a placebo. Therefore Ferreira et al. [45]. showed that the sedative stimulus on the acupoint and, secondarily, the action of the laser on the irradiated area were primarily responsible for the analgesic effects on orofacial, muscular, and articular regions. This theory explains why the intraoral musculature, the temporalis muscle, the submandibular and posterior mandibular regions, which did not receive the direct and localized action of the laser, also showed diminution or remission of the algic symptom in the control group.

### Limitations of the study

The study took into account a diverse range of people. Various scales were employed, ranging from the methods for diagnosing TMD to the method for evaluating the effectiveness of acupuncture and laser acupuncture. The population’s age categories are also a drawback because they are extremely diverse and do not take age into account as a confounding factor. Only age has been found to be a variable factor in certain research’ multiple linear regression analyses. As a result, we can say that there isn’t much support for using acupuncture to treat TMD symptoms. It is not possible to draw exhaustive conclusions. Then, more thorough research is needed to prove beyond a shadow of a doubt that acupuncture is beneficial for this reason.

## Conclusion

The results of our comprehensive review show that there is a sufficient evidence to support the use of acupuncture to treat the symptoms of TMD. However, acupuncture studies that address the long-term efficacy or effectiveness of acupuncture are required, and they must have sufficient sample numbers. Furthermore, data suggests that laser acupuncture is a highly successful treatment for TMJ problems. For those for whom conservative therapy was selected, laser acupuncture is a safe, noninvasive, and efficient therapeutic choice since it lessens the chronic pain linked to TMD and has no negative side effects.

## Data Availability

No datasets were generated or analysed during the current study.
